# Efficacy and safety of optical navigation system versus conventional fluoroscopy in sacral neuromodulation electrode implantation: a prospective randomized controlled trial

**DOI:** 10.1186/s12894-026-02198-y

**Published:** 2026-06-04

**Authors:** Zhou Ziqin, Song Xin, Gu Yinjun, Lv Tingting, Fang Weilin, Huang Jin, L v Jianwei

**Affiliations:** 1 School of Gongli Hospital Medical Technology, University of Shanghai for Science and Technology, Shanghai, China; 2 Department of Urology and Andrology, Pudong Gongli Hospital, Shanghai University of Medicine & Health Sciences, Shanghai, China; 3 Department of Urology and Andrology, Renji Hospital, School of Medicine, Shanghai Jiao Tong University, Shanghai, China

**Keywords:** Neurogenic bladder, Sacral neuromodulation, Optical navigation, Randomized controlled trial, Image-guided surgery

## Abstract

**Background:**

Precise Stage I electrode implantation is critical for the success of sacral neuromodulation (SNM); however, conventional fluoroscopy-guided puncture is limited by reliance on bony landmarks and significant radiation exposure. This study evaluated the clinical value of an optical navigation system (ONS) combined with multimodal image fusion for SNM electrode implantation.

**Methods:**

A prospective, single-blind, randomized controlled trial was conducted between March 2024 and June 2025. Eighty patients with refractory lower urinary tract dysfunction (including neurogenic bladder, overactive bladder, and interstitial cystitis/bladder pain syndrome) undergoing SNM were randomly assigned (1:1) to receive either ONS-assisted puncture (experimental group, *n* = 40) or conventional X-ray guidance (control group, *n* = 40). The primary outcome was the conversion rate to Stage II permanent implantation. Secondary outcomes included the number of puncture attempts, puncture time, total operative time, radiation dose, minimum effective voltage, and perioperative complications.

**Results:**

Baseline characteristics were comparable between groups. The Stage II conversion rate was significantly higher in the ONS group than in the control group (87.5% vs. 62.5%, *P* < 0.05). Patients in the ONS group required fewer median puncture attempts [2.0 (2.0, 2.2) vs. 5.0 (4.0, 7.0), *P* < 0.01] and shorter puncture time [7.5 (5.8, 10.2) min vs. 16.0 (12.0, 25.0) min, *P* < 0.01]. Intraoperative radiation exposure was substantially reduced in the ONS group [145.5 (108.4, 202.3) mGy vs. 473.3 (354.5, 635.2) mGy, *P* < 0.01]. Furthermore, the minimum effective voltage was significantly lower in the ONS group [1.8 (1.8, 2.5) V vs. 2.8 (1.8, 3.0) V, *P* = 0.010], suggesting superior electrode-neural positioning accuracy. No surgical complications occurred in either group.

**Conclusions:**

ONS combined with multimodal image fusion significantly improves the precision of SNM electrode implantation, reduces surgical trauma and radiation exposure, and increases Stage II conversion rates. This technique demonstrates stable clinical efficacy across various etiologies of refractory lower urinary tract dysfunction and represents a valuable navigation tool warranting broader clinical adoption.

**Trial registration:**

Chinese Clinical Trial Registry #ChiCTR2500098093 3/3/2025.

## Background

Sacral neuromodulation (SNM) has emerged as an effective minimally invasive therapy for refractory lower urinary tract dysfunction (LUTD) via electrical stimulation of the S2–S4 sacral nerve roots [1]. The procedure is typically performed in two stages, wherein the precision of Stage I electrode implantation represents a critical determinant of therapeutic success. Suboptimal lead placement compromises the number of effective contacts, increases stimulation thresholds, and reduces implantable pulse generator longevity [2, 3]. However, conventional fluoroscopy-guided puncture relies heavily on bony landmarks and surgeon experience, often proving inadequate in patients with sacral anatomical variations, obesity, or intraoperative positional changes. This “blind” approach frequently necessitates repeated needle adjustments, prolonging operative time and exposing both patients and medical staff to substantial ionizing radiation [4, 5].

To overcome these limitations, various auxiliary navigation techniques have been explored. Computed tomography (CT) guidance provides superior anatomical resolution yet entails significant radiation exposure [6]; ultrasound offers real-time imaging but is constrained by poor penetration through bone cortex [7]; while three-dimensional (3D) printing navigation templates and augmented reality (AR) navigation, despite their potential, remain limited by high costs, complex preoperative preparation, and inability to adapt dynamically to soft tissue deformation [8, 9]. Hence, the search for an auxiliary technique capable of high-precision real-time guidance while minimizing radiation exposure remains a research priority in the field of SNM.

Optical navigation system (ONS) represents a promising alternative that integrates multimodal image fusion (combining CT bony structures with MRI soft-tissue details) with infrared optical tracking to provide real-time, submillimeter-level three-dimensional visualization [10]. This technology has demonstrated reliability in percutaneous nephrolithotomy [11] and orthopedic spine surgery [12], yet its application in SNM has been limited to in vitro phantom studies [13], with no prospective clinical validation conducted to date. Therefore, we conducted this prospective randomized controlled trial to investigate the clinical value of ONS combined with multimodal image fusion in SNM electrode implantation, hypothesizing that this technique would improve implantation precision, reduce radiation exposure, and enhance Stage II conversion rates compared with conventional fluoroscopic guidance.

## Methods and design

### Study design

This study was designed as a prospective, single-blind, randomized controlled trial (RCT). Patients were randomly assigned in a 1:1 ratio to receive either ONS-assisted puncture (experimental group) or conventional X-ray fluoroscopy-guided puncture (control group) in a parallel fashion, with the intent to demonstrate the superiority of ONS guidance in improving electrode implantation precision, reducing radiation exposure, and enhancing Stage II conversion rates compared with conventional fluoroscopy. All procedures were performed between March 2024 and June 2025.

### Study locations

The study took place at Gongli Hospital, Pudong New Area, Shanghai, China, a high-volume academic medical center. The Department of Urology and Andrology serves as a tertiary referral center for complex lower urinary tract dysfunction and pelvic floor disorders, with extensive experience in minimally invasive urological surgery and sacral neuromodulation.

### Study population and recruitment

**Fig. 1:**
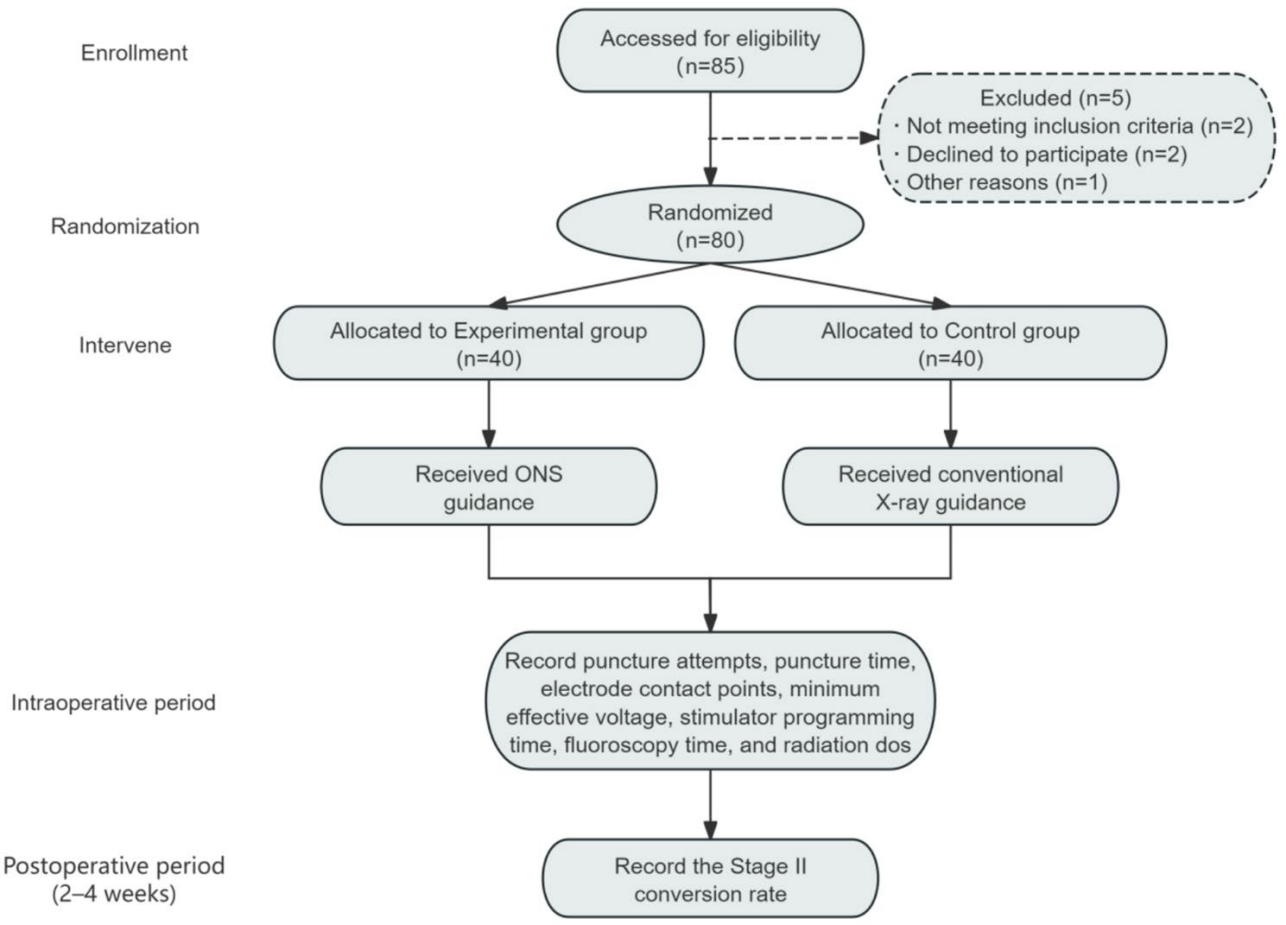
CONSORT flow diagram. Optical navigation system (ONS)

Patients were initially identified through outpatient urology clinics with the assistance of attending physicians according to the inclusion and exclusion criteria detailed below. All consecutive eligible patients were approached for enrollment. Between March 2024 and June 2025, we screened 85 patients scheduled for SNM therapy. Of these, 5 were excluded (2 due to severe uncontrolled urinary tract infection, 2 declined participation, and 1 for other reasons), resulting in 80 eligible patients who were enrolled and randomly assigned (Fig. 1).

#### Inclusion criteria

- Aged 18–75 years, either sex;
- Clinical diagnosis consistent with the Chinese Expert Consensus on Clinical Application of Sacral Neuromodulation or expanded indications, specifically including neurogenic bladder (NB), refractory overactive bladder (OAB), and interstitial cystitis/bladder pain syndrome (IC/BPS) [14], with documented failure or intolerance to conservative treatments (e.g., behavioral therapy, pharmacotherapy);
- Completion of systematic preoperative evaluation, including detailed medical history, physical examination, laboratory tests, and urodynamic studies;
- Imaging requirements: Preoperative thin-slice CT of the sacrococcygeal region and lower abdominal magnetic resonance imaging (MRI) with clear, complete datasets suitable for three-dimensional reconstruction and registration in the optical navigation system;
- Voluntary participation with provision of written informed consent.

#### Exclusion criteria

- Severe, uncontrolled urinary tract infection;
- Confirmed urological malignancy or severe pelvic anatomical abnormality;
- Definitive bladder outlet obstruction;
- Pregnancy or lactation;
- Severe psychiatric disorders, cognitive impairment, or poor compliance precluding completion of scheduled follow-up visits.

### Randomization and blinding

Randomization was performed using a computer-generated random number sequence. The allocation schedule was placed in sequentially numbered, opaque, sealed envelopes and maintained by a research assistant independent of the surgical team and outcome assessors. Following confirmation of eligibility and obtaining informed consent, the envelope was opened to determine group assignment.

Due to the nature of the surgical intervention, the operating surgeons could not be blinded. To minimize performance and detection bias, strict patient-blinding and assessor-blinding were implemented. Patients remained unaware of their group allocation throughout the preoperative, intraoperative, and follow-up periods. Research personnel responsible for postoperative data collection and the statistician performing the final analysis were kept blinded to treatment assignment.

### Intervention

All SNM procedures were performed by a single team of experienced urological surgeons under local anesthesia using the InterStim System (Medtronic, Minneapolis, MN, USA). The procedure comprised two stages: Stage I (test stimulation) and Stage II (permanent implantation). Patients proceeded to Stage II if they demonstrated ≥ 50% improvement in symptoms or high subjective satisfaction during the test phase. Prophylactic antibiotics were routinely administered postoperatively to prevent infection.

#### Control group

Patients underwent conventional fluoroscopy-guided puncture. The S3 sacral foramen was localized using standard bony landmarks under anteroposterior and lateral X-ray guidance, followed by tined lead implantation.

#### Experimental group

**Fig. 2:**
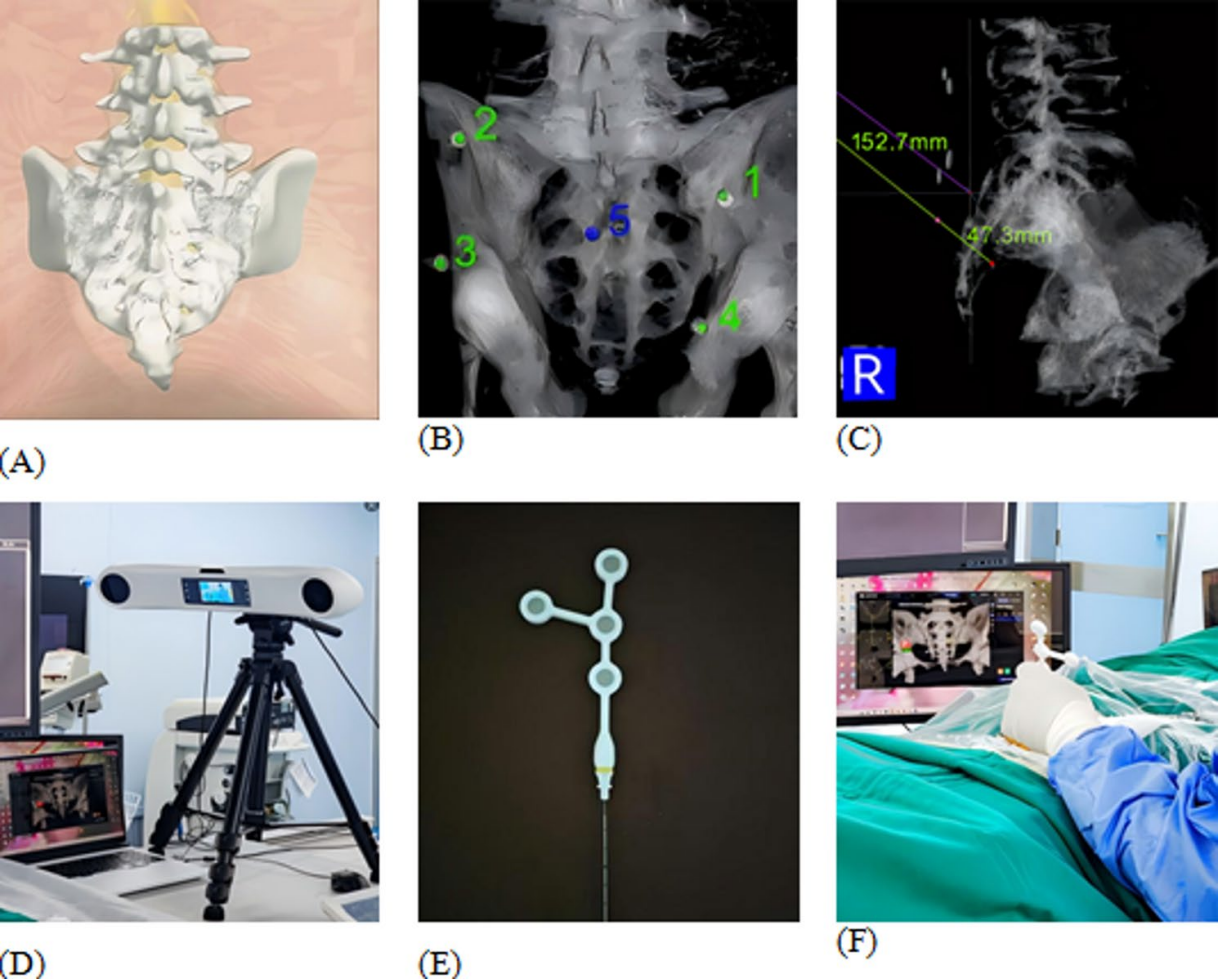
Optical navigation system (ONS) setup and multimodal image fusion. **A** Multimodal fusion of CT and MRI images. **B** Placement of five adhesive skin markers. **C** Manual trajectory planning. **D** The ONS cart positioned adjacent to the patient. **E** Needle cap with optical reflective spheres attached to the sacral nerve puncture needle. **F** Real-time needle puncture guided by ONS

Using the same InterStim electrode model (Medtronic) as the control group. Within 24 h preoperatively, five adhesive radio-opaque skin markers were securely placed on the skin surface surrounding the intended puncture region. Patients subsequently underwent thin-slice CT of the sacrococcygeal region and lower abdominal MRI in the prone position to capture the natural anatomical state of the sacrum and sacral nerves. Imaging data were processed using the SpineNavigation software (version tv1.0.1; Guangdong Aimooe Medical Technology Co., Ltd., Guangdong, China) to perform multimodal image fusion primarily based on sacral bony landmarks and trajectory planning. The software registered CT and MRI datasets to automatically segment and reconstruct three-dimensional models of the sacral bony architecture and sacral nerve root trajectories, displaying the sacral foramina and nerve roots simultaneously. Surgeons manually selected the skin entry point and target point within the sacral foramen to generate a virtual puncture trajectory. Preoperative preparation and surgery were performed separately. Intraoperatively, the optical navigation system cart (equipped with binocular cameras) and control system cart were positioned adjacent to the patient. A needle cap equipped with optical reflective spheres was fitted to the sacral nerve puncture needle. By sequentially contacting the centers of the five preoperatively placed skin markers, needle registration and spatial calibration were completed within the navigation software. Subsequently, the system tracked the spatial transformation via binocular localization to display the puncture needle tip position in real time. The needle was then advanced along the predetermined trajectory to the target (Fig. 2).

### Outcomes

#### Primary outcome measure

The number of puncture attempts, defined as the total number of needle insertions required from initial skin penetration to confirmation of satisfactory electrode position.

#### Secondary outcomes

Secondary outcomes.

1. Technical parameters:
  Puncture time (minutes), defined as the duration from initial skin contact of the puncture needle to successful passage through the sacral foramen and arrival at the S3 target region.
  Total operative time (minutes), recorded from the completion of patient positioning and sterile draping to final electrode fixation and wound dressing.
  Fluoroscopy time (minutes) and cumulative radiation dose (milligrays, mGy), representing the total C-arm X-ray exposure duration and accumulated radiation exposure to the patient during the procedure, respectively.
  Minimum effective voltage (Volts, V), defined as the lowest stimulation amplitude capable of consistently eliciting standard sensory (e.g., perineal paresthesia) or motor (e.g., bellows response, toe flexion) responses during intraoperative electrophysiological testing.
  Number of effective electrode contact points (of four), defined as the count of contacts on the quadripolar lead capable of eliciting valid responses at low stimulation thresholds.
  Stimulator programming time (minutes), measured from successful lead placement to completion of impedance testing and determination of response thresholds across all contact combinations to establish a satisfactory stimulation program.
  Estimated intraoperative blood loss (milliliters, mL) and supplemental anesthesia rate, defined as the proportion of patients requiring additional local anesthetic or intravenous analgesia due to intolerable puncture-related pain.

(2) Safety and conversion metrics: Stage II conversion rate and perioperative complications (infection, hematoma, electrode displacement, etc.).

### Sample size calculation

The sample size was calculated based on the primary outcome (number of puncture attempts). Referencing data from the in vitro model study by Moreta-Martínez et al. [13] (reporting 3.65 ± 1.2 attempts for conventional fluoroscopy vs. 1.23 ± 0.8 for ONS navigation) and our center’s preliminary pilot data (*n* = 10 per group, mean 4.8 ± 1.6 vs. 1.9 ± 0.9 attempts, respectively), we assumed a mean of 5.0 attempts for the control group and 2.0 for the experimental group, accounting for anticipated increased variability due to complex anatomical conditions (e.g., sacral deformities, obesity). The effect size (Cohen’s d = 0.75) was conservatively selected based on our pilot data (calculated d ≈ 2.23) to reflect anticipated greater variability in clinical practice, representing a large effect (approaching Cohen’s threshold of 0.8) and a clinically meaningful ~ 60% reduction in attempts. With a two-sided α of 0.05 and power (1-β) of 80%, 29 patients per group were required. Accounting for 20% dropout, the adjusted sample size was 29 ÷ 0.8 ≈ 37 per group, rounded to 40 per group (total *n* = 80) for operational convenience.

### Statistical analysis

Data were analyzed using SPSS version 27.0 (IBM Corp., Armonk, NY, USA). The Kolmogorov-Smirnov test was used to assess the normality of distribution for continuous variables. Normally distributed data were expressed as mean ± standard deviation and compared between groups using independent samples t-test. Non-normally distributed data are presented as median (Q1, Q3) and analyzed using the Mann-Whitney U test. Categorical variables were expressed as n (%) and compared using Chi-squared test or Fisher’s exact test when expected frequencies were < 5. A two-tailed P-value ≤ 0.05 was considered statistically significant.

### Ethics and dissemination

This study was approved by the Ethics Review Committee of Gongli Hospital (approval number: 2024-076) and conducted in accordance with the Declaration of Helsinki. Written informed consent was obtained from all participants prior to enrollment. This trial was performed in accordance with the Consolidated Standards of Reporting Trials (CONSORT) guidelines [15] and Good Clinical Practice (GCP) standards. Handling of all personal data strictly complied with the Personal Information Protection Law of the People’s Republic of China. The trial was registered prospectively in the Chinese Clinical Trial Registry (ChiCTR) (registration number: ChiCTR2500098093; registration date: March 3, 2025).

## Results

### Participant characteristics

Of the 85 patients assessed for eligibility, 5 were excluded (2 due to severe urinary tract infection, 2 declined participation, and 1 for other reasons), resulting in 80 patients randomly assigned to the ONS group (*n* = 40) or the control group (*n* = 40). Baseline characteristics were well balanced between the two groups: the experimental group comprised 18 men and 22 women with a mean age of 57.5 ± 14.8 years and a mean BMI of 23.9 ± 2.9 kg/m²; the control group comprised 24 men and 16 women with a mean age of 56.4 ± 16.0 years and a mean BMI of 23.3 ± 2.8 kg/m². No statistically significant differences were observed between groups in age, sex, BMI, comorbidities, clinical diagnosis (NB, OAB, IC/BPS), history of lumbosacral surgery, or sacrococcygeal deformities (*P* > 0.05, Table 1).

**Table 1:** Comparison of baseline clinical characteristics

Characteristic	Experimental Group (*n* = 40)	Control Group (*n* = 40)	Statistic	*P* value
Age (years)	57.5 ± 14.8	56.4 ± 16.0	t =−0.32	0.748
BMI (kg/m²)	23.9 ± 2.9	23.3 ± 2.8	t = 0.89	0.375
Gender [n (%)]			χ² = 0.20	0.654
Male	18 (45.0)	24 (60.0)		
Female	22 (55.0)	16 (40.0)		
Diabetes [n (%)]	10 (25.0)	8 (20.0)	χ² = 0.29	0.591
Clinical diagnosis [n (%)]			χ² = 0.63	0.729
Neurogenic bladder	25 (62.5)	28 (70.0)		
Overactive bladder	10 (25.0)	9 (22.5)		
Interstitial cystitis/Bladder pain syndrome	5 (12.5)	3 (7.5)		
History of spine surgery [n (%)]	9 (22.5)	7 (17.5)	χ² = 0.31	0.576
Sacral deformity [n (%)]	11 (27.5)	9 (22.5)	χ² = 0.27	0.603

### Efficacy and safety outcomes

All patients successfully underwent Stage I test stimulation. The Stage II conversion rate was significantly higher in the ONS group than in the control group (87.5% vs. 62.5%, *P* < 0.05). Compared with the control group, the ONS group demonstrated significantly fewer intraoperative puncture attempts, shorter puncture time, reduced fluoroscopy time and radiation exposure, shorter total operative time, and less intraoperative blood loss. Additionally, the ONS group required less supplemental anesthesia during the procedure. Although no statistically significant difference was observed in the number of effective electrode contact points between groups, the ONS group exhibited lower minimum effective voltage and shorter stimulator programming time. All patients were discharged within two postoperative days without any surgery-related complications such as incisional infection or subcutaneous hematoma ( Table 2).

**Table 2:** Comparison of intraoperative metrics during sacral neuromodulation lead implantation

Indicators	Experimental Group (*n* = 40)	Control Group (*n* = 40)	Z Value	*P* value
Puncture attempts [M(Q1,Q3), times]	2.0 (2.0, 2.2)^†^	5.0 (4.0, 7.0)	−7.25	< 0.01
Puncture time [M(Q1,Q3), min]	7.5 (5.8, 10.2)	16.0 (12.0, 25.0)	−6.00	< 0.01
Total operative time [M(Q1,Q3), min]	51.5 (48.0, 57.0)	68.0 (62.0, 74.0)	−6.56	< 0.01
Electrode contact points [M(Q1,Q3), n]	3.0 (3.0, 4.0)	3.0 (2.8, 3.2)	−1.01	0.261
Minimum effective voltage [M(Q1,Q3), V]	1.8 (1.8, 2.5)	2.8 (1.8, 3.0)	−2.38	0.010
Stimulator programming time [M(Q1,Q3), min]	9.5 (8.0, 11.0)	16.0 (13.0, 20.0)	−6.62	< 0.01
Fluoroscopy time [M(Q1,Q3), min]	1.5 (1.1, 2.1)	4.5 (3.6, 5.8)	−7.03	< 0.01
Radiation dose [M(Q1,Q3), mGy]	145.5 (108.4, 202.3)	473.3 (354.5, 635.2)	−6.00	< 0.01
Blood loss [M(Q1,Q3), ml]	4.0 (3.8, 5.0)	6.0 (5.0, 7.0)	−5.08	< 0.01
Supplemental anesthesia [n(%)]	5 (12.5)	14 (35.0)	—*	0.034

^†^Q1 = median indicates ≥ 50% of cases achieved the minimum value (right-skewed distribution). *Fisher’s exact test was used for categorical variables; — indicates not applicable

## Discussion

SNM has emerged as an effective minimally invasive therapy for refractory lower urinary tract dysfunction. The procedure is typically performed in two stages: Stage I involves temporary electrode implantation near the sacral nerves to assess therapeutic efficacy, with progression to Stage II permanent implantation contingent upon ≥ 50% symptom improvement or satisfactory subjective response. Previous studies have established that the long-term success of Stage II implantation is intimately linked to electrode positioning accuracy. Specifically, the number of effective electrode contacts and the minimum effective voltage have been recognized as critical predictors of treatment success [16]. Tilborghs et al. [17] demonstrated that even minor deviations in lead placement significantly increase puncture failure rates, prolong operative time, and reduce the proportion of programmable effective contacts, thereby shortening implantable pulse generator longevity. Superior electrode-nerve interface proximity yields enhanced sensory and motor responses, providing greater flexibility for postoperative parameter optimization. These mechanisms underscore that precise anatomical coupling between the puncture needle and sacral neural structures constitutes the cornerstone of SNM success. However, conventional fluoroscopy-guided techniques rely predominantly on bony landmarks and surgeon experience, often proving inadequate in scenarios involving sacral developmental anomalies, obesity, or intraoperative positional changes. The resultant repeated fluoroscopic adjustments not only extend procedural duration but also expose patients and medical staff to substantial ionizing radiation risks. These limitations have motivated the exploration of auxiliary navigation technologies capable of providing real-time, precise guidance with minimal radiation exposure.

To overcome these constraints, various adjunctive navigation techniques have been developed, each possessing distinct limitations. Meissnitzer et al. [18] reported CT-guided puncture approaches that provide clear visualization of osseous foraminal anatomy and facilitate needle angulation assessment, thereby improving placement precision. Nevertheless, CT guidance entails significant radiation exposure for both patients and operators, and its limited soft-tissue resolution precludes simultaneous functional and anatomical targeting. Ultrasound guidance [7] offers radiation-free real-time imaging but is constrained by poor acoustic penetration through bone cortex, resulting in inadequate visualization of deep sacral foraminal structures and frequently necessitating secondary fluoroscopic confirmation; moreover, this technique demands considerable operator expertise in ultrasonography. 3D printing navigation templates [19], constructed from preoperative CT/MRI data, may reduce the learning curve for novice surgeons, and Liu et al. [16] have reported promising applications of 3D printing combined with ultrasound in patients with pelvic structural anomalies. However, such templates represent static navigation systems incapable of compensating for intraoperative soft-tissue deformation or positional changes; additionally, their fabrication requires substantial time and financial resources, rendering them impractical for emergency or high-throughput elective procedures. AR technology [20] superimposes virtual images onto the operative field to enable real-time three-dimensional trajectory navigation. Despite its theoretical advantages, AR systems demonstrate registration accuracy degradation secondary to intraoperative positional changes, require specialized technical support for calibration, and involve substantial hardware costs, with complex preoperative preparation potentially prolonging hospitalization and increasing healthcare expenditures. Collectively, while existing navigation modalities offer partial solutions, clinical practice continues to lack an optimal strategy that simultaneously provides real-time guidance, minimizes radiation exposure, and streamlines workflow. Accordingly, the present study innovatively applied multimodal image fusion integrated with ONS technology to SNM electrode implantation.

ONS technology utilizes infrared optical tracking combined with multimodal image fusion, employing high-frame-rate binocular cameras to capture spatial coordinates of retroreflective markers and achieve submillimeter registration with preoperative CT/MRI datasets to construct dynamic three-dimensional navigation interfaces [21]. This technology enables real-time visualization of surgical instrument-anatomy relationships and has been extensively validated in neurosurgical and orthopedic procedures requiring high precision [22]. The present study further elucidates multiple clinical advantages of ONS in SNM procedures:

1. Significant optimization of surgical efficiency and safety: Consistent with the in vitro findings of Moreta-Martínez et al. (ONS: 1.23 attempts vs. conventional: 3.65 attempts), our clinical data demonstrate that ONS significantly reduced median puncture attempts compared with the control group [2.0 (2.0, 2.2) vs. 5.0 (4.0, 7.0), P<0.01]. Furthermore, by repurposing CT/MRI data acquired during routine preoperative evaluation, the system achieved substantial reductions in intraoperative radiation exposure [145.5 (108.4, 202.3) mGy vs. 473.3 (354.5, 635.2) mGy, P<0.001] without imposing additional imaging burdens on patients, thereby enhancing procedural safety while maximizing the value of existing diagnostic resources.
2. Enhanced implantation quality through precise trajectory planning: Leveraging multimodal image fusion capabilities, ONS enables personalized trajectory customization to optimize electrode-target proximity. Our findings revealed significantly lower minimum effective voltages in the ONS group compared with the control group [1.8 (1.8, 2.5) V vs. 2.8 (1.8, 3.0) V, P=0.010], indicating superior electrode-nerve fiber interface proximity.
3. Streamlined workflow efficiency: Compared with 3D printing (requiring several days for model fabrication) or AR navigation (complex preoperative calibration), ONS requires only preoperative imaging within 24 hours and intraoperative registration requiring approximately 10 minutes. From a patient benefit perspective, this approach avoids prolonged preoperative preparation and reduces extended hospitalization and excessive additional costs associated with specialized equipment.

This study has several limitations. Although we employed a prospective randomized controlled design, the follow-up duration was limited to 6 months, precluding assessment of long-term electrode stability, sustained clinical efficacy, and implantable pulse generator longevity. Additionally, while randomization ensured balanced distribution of diagnostic categories (NB, OAB, and IC/BPS; *P* = 0.729, Table 1) and comparable baseline BMI between groups, the limited sample size precluded meaningful stratified analysis by specific etiology or obesity status. Furthermore, the current ONS implementation focuses primarily on needle trajectory guidance, whereas optimal SNM outcomes depend on additional complex parameters including final electrode depth and contact distribution within the sacral canal. Comprehensive navigation encompassing the entire implantation process represents the focus of our subsequent research endeavors. Future large-scale, multicenter studies with extended follow-up are warranted to further stratify outcomes by specific diagnostic categories and BMI subgroups, thereby establishing more targeted guidance for clinical practice.

This study demonstrated that optical navigation system combined with multimodal image fusion, through real-time guidance and dynamic trajectory optimization, reduces intraoperative puncture attempts, operative time, and radiation exposure while improving Stage II conversion rates in sacral neuromodulation. This technique represents a valuable adjunct to enhance electrode implantation accuracy and procedural safety.

## Data Availability

The datasets used and/or analyzed during the current study are available from the corresponding author on reasonable request.

## Abbreviations

3D: Three-dimensional
AR: Augmented reality
ChiCTR: Chinese Clinical Trial Registry
CONSORT: Consolidated Standards of Reporting Trials
CT: Computed tomography
GCP: Good Clinical Practice
IC/BPS: Interstitial cystitis/bladder pain syndrome
IQR: Interquartile range
ITT: Intention-to-treat
LUTD: Lower urinary tract dysfunction
MRI: Magnetic resonance imaging
NB: Neurogenic bladder
OAB: Overactive bladder
ONS: Optical navigation system
PP: Per-protocol
RCT: Randomized controlled trial
SNM: Sacral neuromodulation

## References

[1] Ginsberg DA, Boone TB, Cameron AP, et al. The AUA/SUFU Guideline on Adult Neurogenic Lower Urinary Tract Dysfunction: Diagnosis and Evaluation. J Urol. 2021;206(5):1097–105. 34495687 10.1097/JU.0000000000002235

[2] Panicker JN, Fowler CJ, Kessler TM. Lower urinary tract dysfunction in the neurological patient: clinical assessment and management. Lancet Neurol. 2015;14(7):720–32. 26067125 10.1016/S1474-4422(15)00070-8

[3] Averbeck MA, Moreno-Palacios J, Aparicio A. Is there a role for sacral neuromodulation in patients with neurogenic lower urinary tract dysfunction? Int Braz J Urol. 2020;46(6):891–901. 32758301 10.1590/S1677-5538.IBJU.2020.99.10 PMC7527110

[4] Huang H, Zeng W, Fan F, Li K, Xu K, Huang H. Radiologically anatomic measurement analysis for the third sacral foramen and an efficient implantation protocol for sacral neuromodulation. Neurourol Urodyn. 2022;41(5):1149–56. 35438814 10.1002/nau.24933

[5] Luchristt D, Amundsen CL. Strategies for difficult fluoroscopic landmarking during sacral neuromodulation lead placement. Urology. 2023;174:218–20. 36638971 10.1016/j.urology.2022.12.029

[6] Shan S, Zhu W, Zhang G, et al. Video-urodynamics efficacy of sacral neuromodulation for neurogenic bladder guided by three-dimensional imaging CT and C-arm fluoroscopy: a single-center prospective study. Sci Rep. 2022;12(1):16306. 36175471 10.1038/s41598-022-20731-5 PMC9521554

[7] Chen Q, Chen G, He X, et al. Application of ultrasound during electrode implantation for sacral neuromodulation in patients with neurogenic bladder secondary to spinal cord disease: a retrospective study. Int Urol Nephrol. 2021;53(7):1325–30. 33743121 10.1007/s11255-021-02824-8

[8] Cui Z, Wang Z, Ye G, Zhang C, Wu G, Lv J. A novel three-dimensional printed guiding device for electrode implantation of sacral neuromodulation. Colorectal Dis. 2018;20(1):O26–9. 29110390 10.1111/codi.13958

[9] Wang VL, Cheng LG, Zhu TS, Patnaik S, Tarin T. Current applications and limitations of augmented reality in urological surgery: a practical primer and ‘state of the field’. Curr Urol Rep. 2025;26(1):56. 40643724 10.1007/s11934-025-01283-3

[10] Zhao J, Tang H, Xu R, Chen G. Intraoperative application of different imaging techniques in sacral neuromodulation: a systematic review and meta-analysis. BMC Urol. 2025;25(1):201. 40804625 10.1186/s12894-025-01903-7 PMC12344920

[11] Spenkelink IM, Zhu X, Fütterer JJ, Langenhuijsen JF. Feasibility of stereotactic optical navigation for needle positioning in percutaneous nephrolithotomy. World J Urol. 2024;42(1):181. 38507097 10.1007/s00345-024-04870-0 PMC10954992

[12] Yao Q, Wei Y, Wang M, et al. Analysis of the efficacy of Endo-TLIF and OLIF-360 assisted by optical navigation in the treatment of degenerative lumbar spondylolisthesis lesions: a retrospective cohort study. Spine J. S1529-9430(26)00015-X. 10.1016/j.spinee.2026.01.015 41525955

[13] Moreta-Martínez R, Rubio-Pérez I, García-Sevilla M, et al. Evaluation of optical tracking and augmented reality for needle navigation in sacral nerve stimulation. Comput Methods Programs Biomed. 2022;224:106991. 35810510 10.1016/j.cmpb.2022.106991

[14] Chinese Urological Association, Continence Society. Chinese expert consensus on the clinical application of sacral neuromodulation (third edition). Chin J Urol. 2024;45(09):649–53.

[15] Schulz KF, Altman DG, Moher D, Group CONSORT. CONSORT 2010 statement: updated guidelines for reporting parallel group randomised trials. BMJ. 2010;340:c332. 20332509 10.1136/bmj.c332 PMC2844940

[16] Liu R, Zhou Y, Hao Q, et al. Effectiveness of sacral neuromodulation with 3D printing and ultrasound localization for treating neurogenic bladder in patients with pelvic structural anomalies. Asian J Surg. 2025;48(2):1022–6. 10.1016/j.asjsur.2024.10.153 39537485

[17] Tilborghs S, Van de Borne S, Vaganée D, Fransen E, De Wachter S. A Deep Analysis of the Pelvic Floor Motor Response in Sacral Neuromodulation Linking It to Outcome. Neuromodulation. 2025;28(5):787–95. 10.1016/j.neurom.2024.09.477 39580744

[18] Meissnitzer T, Trubel S, Posch-Zimmermann R, Meissnitzer MW. CT-Guided Lead Placement for Selective Sacral Neuromodulation to Treat Lower Urinary Tract Dysfunctions. AJR Am J Roentgenol. 2015;205(5):1139–42. 26496564 10.2214/AJR.14.14270

[19] Gu Y, Lv T, Jiang C, Lv J. Neuromodulation of the Pudendal Nerve Assisted by 3D Printed: A New Method of Neuromodulation for Lower Urinary Tract Dysfunction. Front Neurosci. 2021;15:619672. 33716649 10.3389/fnins.2021.619672 PMC7952533

[20] Yuan H, Xiao Y, Lin X, et al. Application of Augmented Reality for Accurate Punctures During Stage 1 Sacral Neuromodulation. Int Neurourol J. 2024;28(4):302–11. 39765343 10.5213/inj.2448330.165 PMC11710959

[21] Scarone P, Chatterjea A, Jenniskens I, et al. Percutaneous thoraco-lumbar-sacral pedicle screw placement accuracy results from a multi-center, prospective clinical study using a skin marker-based optical navigation system. Eur Spine J. 2022;31(11):3098–108. 36149493 10.1007/s00586-022-07387-5

[22] Faraj MK, Kailan SL, Al-Neami AQH. A New Simple, Cost-Effective Navigation System (EASY Navigator) for Neurosurgical Interventions. World Neurosurg. 2022;164:143–7. 35490891 10.1016/j.wneu.2022.04.100

